# Accelerating next generation sequencing data analysis: an evaluation of optimized best practices for Genome Analysis Toolkit algorithms

**DOI:** 10.5808/GI.2020.18.1.e10

**Published:** 2020-03-31

**Authors:** Karl R. Franke, Erin L. Crowgey

**Affiliations:** Department of Pediatrics, Nemours Alfred I duPont Hospital for Children, Wilmington, DE 19803, USA

**Keywords:** clinical genomics, Genome Analysis Toolkit, GPUs, next generation sequencing, variant detection

## Abstract

Advancements in next generation sequencing (NGS) technologies have significantly increased the translational use of genomics data in the medical field as well as the demand for computational infrastructure capable processing that data. To enhance the current understanding of software and hardware used to compute large scale human genomic datasets (NGS), the performance and accuracy of optimized versions of GATK algorithms, including Parabricks and Sentieon, were compared to the results of the original application (GATK V4.1.0, Intel x86 CPUs). Parabricks was able to process a 50× whole-genome sequencing library in under 3 h and Sentieon finished in under 8 h, whereas GATK v4.1.0 needed nearly 24 h. These results were achieved while maintaining greater than 99% accuracy and precision compared to stock GATK. Sentieon’s somatic pipeline achieved similar results greater than 99%. Additionally, the IBM POWER9 CPU performed well on bioinformatic workloads when tested with 10 different tools for alignment/mapping.

## Introduction

As the price of next generation sequencing (NGS) decreases and the data footprint increases, compute power is a major limitation. The bioinformatics processing of NGS data is routine in many translational research and precision medicine efforts with the Genome Analysis Toolkit (GATK) from the Broad Institute and their “Best Practices” workflows [1-3] being widely accepted as standards for germline and somatic short variant discovery.

The majority of computational algorithms specific for NGS were developed for use in a CPU environment with standard threading techniques. However, as NGS becomes more routine, the need to offer faster processing is essential. Despite the fact that x86_64 (henceforth x86) CPUs from Intel represent the most common processing architecture in both the server space as well as desktop workstations, alternative architectures exist which offer some advantages such as POWER9 from IBM and GPUs from NVIDIA.

This article compares the speed, accuracy, and scalability of the official GATK algorithms running on Intel x86 CPUs, a POWER9 optimized version provided by IBM, a GPU optimized version provided by Parabricks, and an x86 optimized version provided by Sentieon [4]. Additionally, the ability of POWER9 CPUs to handle bioinformatic workloads is assessed by comparing the performance of a number of other bioinformatic tools compiled to run on both x86 and POWER9 systems.

## Methods

### Hardware configuration

All x86 jobs were run on an HPC cluster node (Thinkmate HDX XB24-5260V4) powered by two 8-core Intel Xeon E5-2620 v4 CPUs and 256GB ECC memory. All POWER9 jobs were run on an HPC cluster node (IBM AC922) powered by two 16-core POWER9 CPUs (Part #02CY417), 512GB ECC memory, and four Nvidia TESLA V100 GPUs each with 32GB HBM2 memory. Disk I/O was handled via a DDN storage appliance connected to each node via InfiniBand.

### Software configuration

Job scheduling on the HPC cluster was handled by SLURM. GATK software was run with parameters according to their best practices workflows with performance optimizations found by Heldenbrand et al. [5] with any deviations indicated as such. The Sentieon and Parabricks pipelines were run according to their included documentation. Runtimes were calculated using the GNU time utility.

### Sensitivity and precision calculations

GATK’s Concordance tool was utilized to compare the tested Variant Call Format (VCF) outputs to different reference VCFs depending on the comparison being made. The reference VCF is treated as a truthset, and the number of true-positive, false-positive, and false-negative variants in the query VCF are counted. Sensitivity is calculated as true-positives/(true-positives + false-negatives). Precision is calculated as true-positives/(true-positives + false-positives).

### POWER9 testing workloads

For the RNA sequencing (RNAseq) benchmarks with STAR v2.7.0c [6], Tophat v2.1.1 [7], HISAT2 v2.1.0 [8], and BBMap v38.34 [9], 15 paired end RNAseq libraries were downloaded from the ENA database (Study PRJEB23554). FASTQ files trimmed via BBDuk before being mapped to GRCh38 using each tool’s default settings. For BBMap and Tophat only one library was used for benchmarking, while HISAT2 and STAR performed mapping using all 15. For the splice aware aligners, STAR, HiSat2, and Tophat, the Gencode v29 annotation GTF file was used.

For the DNA short read mapping benchmarks with BWA MEM v0.7.17-r1188 [10], Bowtie v1.2.2 [11], and Bowtie2 v2.3.5.1 [12], the NA12878 50x WGS library was used. The FASTQ files were mapped to GRCh38 using each tool’s default settings.

For blastx v2.8.1 [13], all *Brachypodium distachyon* transcript sequences from the BD21 v2.1 annotation were queried against the *Oryza sativa* v7 MSU protein database. For blastn v2.8.1 [13], the same transcript sequences were queried against a local copy of NCBI’s nt database. For pblat v36x2 [14], all human transcript sequences from the Gencode v29 release were used as a query against GRCh38.

## Results

### Germline variant detection pipeline speed and scalability

**Fig. 1.**
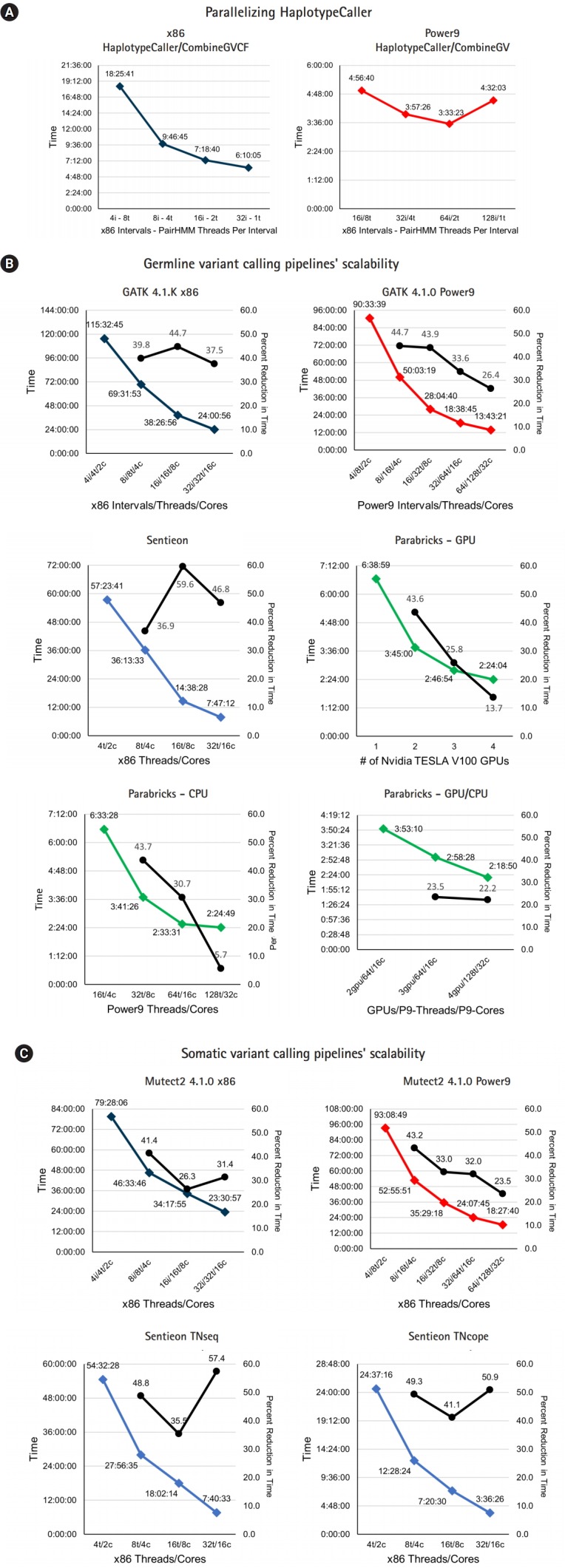
Variant calling pipelines’ scalability. (A) To determine the most efficient way of parallelizing GATK’s HaplotypeCaller, different combinations of scattered intervals and PairHMM OpenMP threads were tested on x86 and Power9 systems. The recorded times also include the CombineGVCFs step. (B) The scalability of GATK’s HaplotypeCaller pipeline on x86 and Power9 was tested with varying amounts of compute resources alongside Sentieon’s DNAseq and Parabricks. (C) Scalability of GATK’s Mutect2 pipeline on x86 and Power9 was tested with varying amounts of compute resources alongside Sentieon’s TNseq and TNscope. At the time of this analysis, Parabricks had not yet ported their somatic pipeline to Power9 therefore it could not be tested.

The pipelines for germline variant discovery were tested using a 50× WGS library from the NA12878 sample of Illumina's Platinum Genomes project (ENA study PRJEB3381) [15]. The baseline run of GATK4 took over 100 h on the x86 system mainly due to the HaplotypeCaller step (Table 1). The most significant speed improvements (Fig. 1A) were demonstrated with single threaded PairHMM and splitting the hg38 genome into 32 scattered intervals (two per x86 core); this also allowed for parallelization of the BaseRecalibrator and ApplyBQSR steps. This manual form of multithreading brought the nearly 107-h runtime down to just under 24 h (Table 1). On the POWER9 with SMT4, the most efficient use of resources was observed when 2 intervals were run per core with each using 2 PairHMM threads (Fig. 1A) which yielded a ~10 h decrease in runtime (–42.4%) compared to x86 with lower times in the BWA, MarkDuplicates, and HaplotypeCaller steps.

The Sentieon DNAseq/DNAscope pipelines reduced runtimes even further to under 8 h with much faster BWA, MarkDuplicates, and HaplotypeCaller steps. Sentieon’s version of BWA finished ~3 h earlier (–38.2%) than stock on the same x86 node. Marking duplicate reads only took 39 minutes whereas previously mentioned pipelines measured that time in hours. Finally, the HaplotypeCaller step took just over 1 h for Sentieon’s pipeline. Nearly identical runtimes were observed for DNAseq and DNAscope pipelines with the latter being Sentieon’s proprietary algorithm and not a reimplementation of HaplotypeCaller. The fastest analysis performed was Parabricks which yielded a genomic VCF (GVCF) file in 2 h 21 min. When set to output VCF directly, with no GVCF conversion, a faster runtime of 2 h 3 min was observed.

Stock GATK did not scale linearly due to having multiple single threaded steps (Fig. 1B); this is in contrast to Sentieon’s implementation which was almost completely multithreaded allowing for much better scalability. In GPU scalability testing, Parabricks hit diminishing returns when adding a fourth GPU which only reduced runtime by ~23 min (–13.7%) whereas going from two to three GPUs reduced the runtime by about an hour (–25.8%).

### Germline variant detection pipeline accuracy

To determine the accuracy of the various analyses, GATK’s concordance tool was utilized to compare the final VCF outputs to GATK4’s baseline VCF and the NA12878 Platinum Genomes truthset VCF (Table 2). Parabricks most closely matched the GATK baseline run with 99.8% accuracy and precision for single nucleotide polymorphisms (SNPs) and 99.1% accuracy and precision for INDELs. When the output was compared with the version of GATK Parabricks was designed to match (v4.0.4), accuracy and precision were both 99.99% (data not shown). Sentieon’s DNAseq pipeline scored 99.7% for accuracy and precision in calling SNPs, but had around one percentage point decrease for INDEL calling.

In the truthset comparison, Sentieon’s DNAscope yielded the highest SNP sensitivity by calling ~7,000 SNPs that the other callers missed; however, this increase in sensitivity comes at the cost of a loss in precision due to an additional ~4,000 false-positive SNPs. Despite Sentieon’s DNAseq pipeline having the lowest sensitivity and precision for INDELs in the previous comparison, here it ranks highest on both fronts due to it calling ~200–300 more true-positive INDELs that the other pipelines missed and having ~500 fewer false-positives.

### Somatic variant detection pipeline speed

To test the performance of the somatic variant calling pipelines, the cancer like mixture of two Genome in a Bottle samples, NA12878 and NA24385, constructed by the Hartwig Medical Foundation and Utrecht Medical Center [16], was utilized. The baseline Mutect2 run took the longest at over 118 h due to the minimally parallelized Mutect2 step. Manually parallelizing this step using scattered intervals allowed for a reduction in total runtime to ~23.5 h on one of our x86 nodes and ~18.5 h on the POWER9 node. Sentieon’s TNseq pipeline reduced this even further to ~7.5 h and their TNscope pipeline was the fastest at ~3.5 h.

Of the steps unique to the Mutect2 “Best Practices” pipeline, Mutect2 and GetPileupSummaries account for over 99% of the total time. While GATK v4.1.0 allows for Mutect2 to be manually parallelized via scattered intervals and a subsequent MergeVCF step, there is no built-in method to merge the output of multiple GetPileupSummaries jobs. This fact hurt the scalability of the pipelines on both x86 and POWER9 systems with Sentieon’s pipelines not only being faster, but also seeing greater reductions in time when given more compute resources (Fig. 1C); however, in GATK v4.1.1 the GatherPileupSummaries tool was introduced which would allow for both steps to be parallelized.

### Somatic variant detection pipeline accuracy

The accuracy of the Mutect2 based pipelines was analyzed in a similar fashion to the assessment of the HaplotypeCaller based pipelines via GATK’s Concordance tool (Table 2). TNseq had a higher amount of variation when compared to Mutect2 v4.1.0 than DNAseq had to HaplotypeCaller v4.1.0; however, when TNseq was compared against the specific version of Mutect2 it was designed to mimic (v4.0.2.1), the results were more similar with accuracy and sensitivity both over 99.5% (data not shown).

### Benchmarking other bioinformatic tools: POWER9 for bioinformatics

To assess how well POWER9 handles bioinformatic workloads the performance of ten commonly used alignment/mapping tools were compared when run on our Intel x86 nodes and the IBM POWER9 node: STAR, BBMap, Tophat, HISAT2, BWA MEM, Bowtie, Bowtie2, blastx, blastn, and pblat a parallelized version of BLAT [17]. Each tool was run using default settings with the same workload on both x86 and POWER9. This is not a comparison of the tools themselves; some similar tools were given different workloads, such as STAR and Tophat, to allow for meaningful runtimes. Each tool was run with the following resource configurations to allow for thread-vs-thread and core-vs-core comparisons: x86-16t/8c, x86-32t/8c, POWER9-16t/4c, and POWER9-32t/8c.

**Fig. 2.**
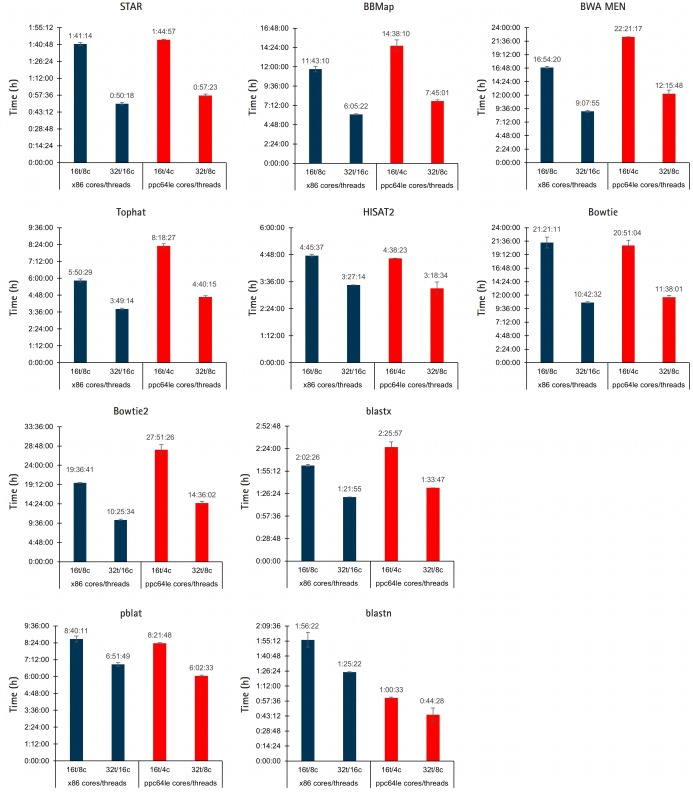
x86/POWER9 performance comparison of aligners/mappers. The performance of 10 different tools for alignment/mapping was compared between POWER9 and ×86 systems. Jobs were run in triplicate across different days; averaged results are shown in the graphs with the error bars representing standard deviation.

For thread-vs-thread comparisons, the results are somewhat split between similar runtimes and a slight edge to x86 threads (Fig. 2). STAR, HISAT2, and Bowtie, achieved nearly identical runtimes on both platforms. BBMap, Tophat, BWA MEM, Bowtie2, and blastx performed better with an equal number of x86 threads, while blastn ran nearly twice as fast with an equal number of POWER9 threads and pBLAT had a slight edge. In the core-vs-core performance comparison, the POWER9 architecture resulted in lower runtimes in every instance.

## Discussion

Both Parabricks and Sentieon offer highly accelerated GATK algorithms that greatly reduce variant calling processing time. Parabricks’s utilization of the higher number of cores available on a GPU compared to a CPU allows for a much faster processing time of a single sample compared to Sentieon. However, the legacy of algorithm development in a CPU environment compared to a GPU for NGS pipelines has impacted the relative number of CPUs available compared to GPUs within an institution. The value of being able to process a single sample in ~2 h should not be discounted, however, especially in the age of precision medicine. Delivering “fast” genome sequencing to help diagnosis a newborn in a neonatal intensive care unit or to identify a somatic variant that can be used in treatment selections is becoming a reality for many institutions.

Our testing of Sentieon’s ability to reproduce GATK’s output yielded similar results to a previous assessment done by Kendig et al. [18]; however, the output from Parabricks was essentially identical to the targeted version which may make it more attractive to those within the research community. If GATK compliance is less of a concern, Sentieon does offer their own proprietary algorithms for variant calling which did perform better when compared to the VCF truthsets used in this study.

While the CPUs used in this comparison are all based on 14nm microarchitectures, the Intel CPUs in our x86 nodes are a few years old, and to do a true x86 versus POWER9 comparison a newer Intel CPU would be needed. Despite this, we felt that these benchmarking comparisons would shed light on POWER9’s ability to handle bioinformatic workloads and prove useful to researchers in designing their compute infrastructure.

The POWER9 architecture performed well in the core-vs-core comparisons. Part of this advantage comes from IBM’s implementation of simultaneous multithreading, which is superior to Intel’s Hyper-threading by allowing for twice as many threads to run simultaneously on the same core. This analysis demonstrated that even in instances where the tools were originally coded to take advantage of Intel specific instruction sets such as SSE2, the total runtime was lower on POWER9 than x86 with identical core counts. However, this does allude to an issue that can arise when using a POWER9 system: not all bioinformatic programs that were originally written for x86 systems can easily compile and run on the POWER9 architecture.

## Figures and Tables

**Table 1.** Variant calling pipelines' speed

	GATK			Sentieon		Parabricks
	Haplotypecaller			DNAseq	DNAscope	
Germline pipeline	x86 baseline	x86 32-interval	Power9 64-interval			
Times						
BWA	8:30:54	8:28:32	4:41:49	5:15:37	5:23:05	1:47:04
MarkDupes	6:35:29	5:35:05	3:05:17	0:39:03	0:38:55	
Samtools Index	1:51:02	1:33:05	1:16:05			
BaseRecalibrator	10:10:20	0:38:20	0:18:01	0:19:34	0:19:45	
ApplyBQSR	7:59:57	0:43:08	0:23:55	0:22:31	0:22:45	0:34:25
HaplotypeCaller	71:02:51	5:30:32	2:28:12	1:05:11	1:03:12	
CombineGVCF	-	0:51:42	0:59:12	-	-	-
GenotypeGVCFs	0:40:38	0:29:11	0:30:50	0:01:12	0:01:16	0:33:13
Total	106:51:11	23:49:35	13:43:21	7:43:08	7:48:58	2:54:42
Somatic pipeline	Mutect2			TNseq	TNscope	N/A
Times						-
Mutect2	109:43:31	14:46:15	9:06:52	7:31:46	3:30:38	-
MergeVCF	-	0:00:30	0:00:44	-	-	-
GetPileupSummaries	8:47:05	8:40:35	9:17:00	0:04:52	-	-
CalculateContamination	0:00:11	0:00:11	0:00:13		-	-
FilterMutectCalls	0:02:22	0:03:26	0:02:51		-	-
Total	118:33:09	23:30:57	18:27:40	7:36:38	3:30:38	-

N/A, not available.

**Table 2.** Variant calling pipelines' accuracy

	Germline				Somatic		
	GATK	Sentieon		Parabricks	GATK	Sentieon	
	Haplotypecaller	DNAseq	DNAscope		Mutect2	TNseq	TNscope
VS Baseline VCF							
SNP							
True-positive	-	3,827,008	3,782,857	3,830,446	-	980,680	1,036,385
False-positive	-	9,703	149,987	7,500	-	28,850	174,668
False-negative	-	11,202	55,353	7,764	-	75,037	19,332
Sensitivity	-	0.99708	0.98558	0.99798	-	0.92892	0.98169
Precision	-	0.99747	0.96186	0.99805	-	0.97142	0.85577
INDEL					-		
True-positive	-	815,205	752,611	818,642	-	67,636	82,766
False-positive	-	11,314	75,083	7,431	-	20,197	71,222
False-negative	-	10,756	73,350	7,319	-	28,441	13,311
Sensitivity	-	0.98698	0.91119	0.99114	-	0.70398	0.86145
Precision	-	0.98631	0.90929	0.99100	-	0.77005	0.53748
VS Truthset VCF							
SNP							
True-positive	3,486,614	3,486,443	3,493,799	3,486,520	827,366	814,549	910,320
False-positive	2,345	2,344	6,541	2,360	983	2,586	9,212
False-negative	108,558	108,729	101,373	108,652	125,113	137,930	42,159
Sensitivity	0.9698	0.96976	0.97180	0.96978	0.86864	0.85519	0.95574
Precision	0.99933	0.99933	0.99813	0.99932	0.99881	0.99684	0.98998
INDEL							
True-positive	548,276	548,574	548,368	548,247	59,112	59,941	82,501
False-positive	9,496	8,987	9,635	9,393	5,654	3,035	12,343
False-negative	24,451	24,153	24,359	24,480	71,354	70,525	47,965
Sensitivity	0.95731	0.95783	0.95747	0.95726	0.45308	0.45944	0.63236
Precision	0.98298	0.98388	0.98273	0.98316	0.9127	0.95181	0.86986

GATK, Genome Analysis Toolkit; VCF, Variant Call Format; SNP, single nucleotide polymorphism.

## References

[1] McKenna A, Hanna M, Banks E, Sivachenko A, Cibulskis K, Kernytsky A (2010). The Genome Analysis Toolkit: a MapReduce framework for analyzing next-generation DNA sequencing data. Genome Res.

[2] DePristo MA, Banks E, Poplin R, Garimella KV, Maguire JR, Hartl C (2011). A framework for variation discovery and genotyping using next-generation DNA sequencing data. Nat Genet.

[3] Van der Auwera GA, Carneiro MO, Hartl C, Poplin R, Del Angel G, Levy-Moonshine A (2013). From FastQ data to high confidence variant calls: the Genome Analysis Toolkit best practices pipeline. Curr Protoc Bioinformatics.

[4] Freed D, Aldana R, Weber JA, Edwards JS The Sentieon Genomics Tools: a fast and accurate solution to variant calling from next-generation sequence data.

[5] Heldenbrand JR, Baheti S, Bockol MA, Drucker TM, Hart SN, Hudson ME Performance benchmarking of GATK3.8 and GATK4.

[6] Dobin A, Davis CA, Schlesinger F, Drenkow J, Zaleski C, Jha S (2013). STAR: ultrafast universal RNA-seq aligner. Bioinformatics.

[7] Kim D, Pertea G, Trapnell C, Pimentel H, Kelley R, Salzberg SL (2013). TopHat2: accurate alignment of transcriptomes in the presence of insertions, deletions and gene fusions. Genome Biol.

[8] Kim D, Langmead B, Salzberg SL (2015). HISAT: a fast spliced aligner with low memory requirements. Nat Methods.

[9] Bushnell B BBMap. SourceForge, 2019. http://www.fda.gov/downloads/Drugs/.../Guidances/UCM174090.pdf.

[10] Li H Aligning sequence reads, clone sequences and assembly contigs with BWA-MEM. http://arxiv.org/abs/1303.3997.

[11] Langmead B, Trapnell C, Pop M, Salzberg SL (2009). Ultrafast and memory-efficient alignment of short DNA sequences to the human genome. Genome Biol.

[12] Langmead B, Salzberg SL (2012). Fast gapped-read alignment with Bowtie 2. Nat Methods.

[13] Altschul SF, Gish W, Miller W, Myers EW, Lipman DJ (1990). Basic local alignment search tool. J Mol Biol.

[14] Wang M, Kong L (2019). pblat: a multithread blat algorithm speeding up aligning sequences to genomes. BMC Bioinformatics.

[15] Eberle MA, Fritzilas E, Krusche P, Kallberg M, Moore BL, Bekritsky MA (2017). A reference data set of 5.4 million phased human variants validated by genetic inheritance from sequencing a three-generation 17-member pedigree. Genome Res.

[16] Cuppen E, Nijman I, Chapman B NA12878/NA24385 tumor-like mixture. NA12878/NA24385 tumor-like mixture. ftp://ftp-trace.ncbi.nlm.nih.gov/giab/ftp/use_cases/mixtures/UMCUTRECHT_NA12878_NA24385_mixture_10052016/.

[17] Kent WJ (2002). BLAT: the BLAST-like alignment tool. Genome Res.

[18] Kendig KI, Baheti S, Bockol MA, Drucker TM, Hart SN, Heldenbrand JR (2019). Sentieon DNASeq variant calling workflow demonstrates strong computational performance and accuracy. Front Genet.

